# Zishen Pill alleviates diabetes in Db/db mice via activation of PI3K/AKT pathway in the liver

**DOI:** 10.1186/s13020-022-00683-8

**Published:** 2022-11-10

**Authors:** You Wu, Boju Sun, Xiaoyuan Guo, Lili Wu, Yaomu Hu, Lingling Qin, Tao Yang, Mei Li, Tianyu Qin, Miao Jiang, Tonghua Liu

**Affiliations:** 1 Key Laboratory of Health Cultivation of the Ministry of Education, Beijing University of Chinese Medicine, Beijing, 102488 China; 2 Key Laboratory of Health Cultivation of Beijing, Beijing University of Chinese Medicine, Beijing, 102488 China; 3 School of Life Science, Beijing University of Chinese Medicine, Beijing, 102488 China; 4 Dongfang Hospital, Beijing University of Chinese Medicine, Beijing, 100078 China; 5 Department of Science and Technology, Beijing University of Chinese Medicine, Beijing, 100029 China

**Keywords:** ZiShen Pill, Diabetes, Insulin resistance, Transcriptomics, Glucolipid metabolism

## Abstract

**Background:**

The rising global incidence of type 2 diabetes mellitus (T2DM) highlights a need for new therapies. The Zishen Pill (ZSP) is a traditional Chinese herbal decoction that has previously shown hypoglycemic effects in C57BL/KsJ-db/db mice, although the therapeutic mechanism remains unknown. This study aims to explore the underlying mechanisms of ZSP’s hypoglycemic effects using db/db mice.

**Methods:**

Db/db mice were divided into two groups: model group and ZSP group, while *wt/wt* mice were used as a normal control. ZSP was given to mice by gavage for 40 days. During treatment, blood glucose level and body weight were monitored continuously. Oral glucose tolerance test (OGTT) was performed at day 35. Blood and tissue samples were collected at the end of treatment for further analyses. Mice liver samples were analyzed with mRNA transcriptomics using functional annotation and pathway enrichment to identify potential mechanisms that were then explored with qPCR and Western Blot techniques.

**Results:**

ZSP treatment significantly reduced weight gain and glycemic severity in db/db mice. ZSP also partially restored the glucose homeostasis in db/db mice and increased the hepatic glycogen content. Transcriptomic analyses showed ZSP increased expression of genes involved in glycolysis including *Hk2*, *Hk3*, *Gck* and *Pfkb1*, and decreased expression of *G6pase.* Additionally, the gene and protein expression of phosphoinositide 3-kinase (PI3K)/protein kinase B (AKT) pathway, and *Csf1* and *Flt3* mRNA expression were significantly upregulated in ZSP group.

**Conclusion:**

ZSP treatment reduced the severity of diabetic symptoms in db/db mice. ZSP increased expression of genes associated with glycogen synthesis and glycolysis, and decreased gluconeogenesis via the enhancement of the PI3K/AKT signaling in the liver.

**Supplementary Information:**

The online version contains supplementary material available at 10.1186/s13020-022-00683-8.

## Introduction

The most recent report by the International Diabetes Federation, estimates the global prevalence of diabetes in adults to be over 10.5% in 2021, effecting hundreds of millions of people [1]. Approximately 90% of these global diagnoses are type 2 diabetes mellitus (T2DM), which is typically accompanied by obesity, cardiovascular disease and other metabolic stressors, causing substantial economic burden on global health systems [2–4]. The rising global incidence indicates that present treatments and preventions for diabetes are insufficient, and that new more potent therapies will be needed.

One potential avenue for new treatments is the exploration of ethnopharmacological methods used to treat diabetes [5]. Traditional Chinese Medicine (TCM), has been practiced for more than 2000 years, and is an important complementary therapy in many countries [6, 7]. The ZiShen Pill (ZSP), also known as TongGuan Wan, is a TCM herbal formular reported to have anti-diabetic effects. ZSP is composed of three herbs: *Rhizoma Anemarrhenae*, *Cortex Phellodendri* and *Cinnamomum cassia*, and a previous study demonstrated anti-diabetic and anti-obesity functions in C57BL/KsJ-db/db mice [8]. Our previous study confirmed the hypoglycemic effect of ZSP application and found it effective in treating diabetic nephropathy by alleviating inflammation [9]. Despite continued interest the molecular mechanisms of ZSP’s therapeutic effects remain unclear.

Insulin resistance is a hallmark of T2DM, defined by a decrease of insulin sensitivity in insulin-targeted tissues including the liver, skeletal muscle and adipose tissue [10]. Insulin resistance may lead to dysregulated glucolipid metabolism which manifests as an increased blood glucose level. The liver is one of the most important organs in energy metabolism and plays an important role in both glucose and lipid metabolic processes [11]. The liver regulates glucose homeostasis by controlling glycogen content and hepatic gluconeogenesis [11], and influences the lipid metabolism by de novo lipogenesis and fatty acid oxidation [12]. As such, liver tissue relies heavily on insulin signaling to maintain glucose levels, hepatic tissue is exposed to insulin via the portal vein, making the concentration of insulin in the liver two to three times higher than in the wider circulatory system. Insulin activity in the liver occurs via the phosphoinositide 3-kinase (PI3K)/protein kinase B (AKT) signal cascade to exert various metabolism-regulating functions [13, 14]. PI3K/AKT signalling is essential for cellular physiology [15] with a well-established role in diabetes [16, 17]. In mammals, PI3K exists in 3 classes, with class I PI3K has a variety of functions closely related to metabolism. The catalytic subunit of class I PI3K is encoded by four genes: *pik3ca*, *pik3cb*, *pik3cd* and *pik3cg*. Likewise there are 3 AKT isoforms, AKT1 and AKT2 exist in the liver where they regulate glucose metabolism [14].

In this study, we explore the effects of ZSP on db/db mice, a common animal model for diabetes, and use transcriptomics to investigate the changes in gene expression in treated mice livers to investigate underlying mechanisms for ZSP’s anti-diabetic effects.

## Materials and methods

### Chemicals and reagents

ZSP is composed of three herbs: *Rhizoma Anemarrhenae*, *Cortex Phellodendri* and *Cinnamomum cassia.* Extracts of *Rhizoma Anemarrhenae*, *Cortex Phellodendri* and *Cortex Cinnamomi* (extraction ratio: 10:1) were purchased from Shaanxi Zhongxin Biotechnology (Xi’an, Shaanxi, China). To produce the decoction, the three extracts were mixed at a ratio of 10:10:1, dissolved in distilled water to 0.468 g/mL and thoroughly stirred before gavage.

### Liquid chromatography (LC)-mass spectrometry (MS)/MS analysis

The components of ZSP were detected by LC–MS/MS analysis. LC–MS/MS analysis was conducted using an Agilent 1290 ultra-high performance liquid chromatography system (Agilent Technologies, Santa Clara, CA, US) with a UPCL BEH C18 column (1.7 μm*2.1 mm*100 mm, Waters, Milford, MA, US). 5 μL of ZSP sample was loaded onto the system and flow rate was set at 400 μL/min. The elution program is described in Additional file 1: Table S1. The MS and MS/MS data were obtained by a Q Exactive Focus mass spectrometer (Thermo Fisher Scientific, Bremen, Germany). Mass ranged from 100 to 1500 in each cycle, and the top three of every cycle were screened for further MS/MS data acquisition. The MS/MS data was matched to the database provided by Shanghai BIOTREE biotech Co., Ltd to identify the materials.

### Animals

The animal experiments in this study were approved by the Animal Care and Ethics Committee of Beijing University of Chinese Medicine (approval No. BUCM-4-2019031003-1089). A total number of 14 6 week-old male C57BL/KsJ-db/db mice and 7 C57BL/KsJ-*wt/wt* mice of the same age and gender were purchased from Nanjing Biomedical Research Institute of Nanjing University (Nanjing, China). Mice were kept in individually ventilated cages with free access to food (normal chow diet) and water. The cages were placed in a specific-pathogen-free environment with a standard 12/12 h artificial light/dark cycle.

After 1 week of accommodation, db/db mice were randomly divided into two groups (ZSP group, receiving 3.3 g/kg ZSP by gavage; model group, receiving distilled water by gavage) according to their blood glucose levels and body weights. *Wt/wt* mice were used as normal group (receiving distilled water by gavage). The dosage of ZSP treatment is calculated according to the dosage of human by the body surface area estimation method determined according to our previous study [9]. The treatment lasted for 40 days and mice received normal chow diet throughout. The body weight and food intake were measured every 3 days. Overnight fasting glucose level was measured every 7 days using blood drawn from the tail vein. At the end of treatment, the body length of mice was measured and body mass index (BMI) calculated as follows:

$${\text{BMI}} = {{{\text{body weight}}\left( {\text{g}} \right)} \mathord{\left/ {\vphantom {{{\text{body weight}}\left( {\text{g}} \right)} {\left[ {{\text{body length}}\left( {\text{m}} \right)^{ \wedge } 2} \right]}}} \right. \kern-\nulldelimiterspace} {\left[ {{\text{body length}}\left( {\text{m}} \right)^{ \wedge } 2} \right]}}$$

### Oral glucose tolerance test (OGTT)

OGTT was conducted on mice at day 35 after over-night fasting. All mice were orally administered 1 g/kg body weight of glucose (dissolved in distilled water to 10%w/v). Blood glucose levels were measured at administration and after 30, 60, and 120 min of glucose treatment. The area under curve (AUC) of OGTT was calculated as follows:

$${\text{AUC}} = 0.25 \times {\text{G0}} + 0.5 \times {\text{G30 + 0}}{.75} \times {\text{G60 + 0}}{.5} \times {\text{G120}}$$

(G0, G30, G60 and G120 represent blood glucose at 0, 30, 60, and 120 min, respectively).

### Tissue harvest and histological observation

After 40 days of treatment, mice were sacrificed after anesthesia with isoflurane. Blood was drawn from abdominal aorta and collected in EP tubes, then centrifuged at 3000 rpm for 15 min at 4 °C to obtain serum. Serum was stored at − 20 °C. Liver and adipose tissue were removed and weighed. Tissue samples were snap frozen with liquid nitrogen and stored at − 80 °C.

A slice of each tissue was kept in a 4% paraformaldehyde-PBS solution (Cat.P1110, Solarbio Science & Technology, Beijing, China) for 24 h before being embedded in paraffin. Hematoxylin & eosin (H&E) staining was conducted for morphological observation, oil red O staining and periodic acid-Schiff (PAS) staining was performed to examine the lipid accumulation and glycogen content, respectively.

### Biochemical examinations

Serum lipids including total cholesterol (TC), triglyceride (TG), high-density-lipoprotein (HDL) and low-density-lipoprotein (LDL), as well as hepatic function indicator alanine transaminase (ALT) and aspartate aminotransferase (AST) levels were measured by a chemistry analyzer (AU480, Beckman Coulter, Brea, CA, US). Hepatic glycogen was tested by colorimetric analysis using a commercial kit (Cat. BC0345, Solarbio Science & Technology). Fasting serum insulin levels were measured by enzyme-linked immunosorbent assay (ELISA) using Mouse Ultrasensitive Insulin ELISA kit (Cat. 80-INSMSU-E01, ALPCO Diagnostics, Salem, NH, US). Homeostatic model assessment for insulin resistance (HOMA-IR) was calculated as follows:

$${\text{HOMA}} - {\text{IR}} = {\text{Fast blood glucose level}}\left( {{{{\text{mmol}}} \mathord{\left/ {\vphantom {{{\text{mmol}}} {\text{L}}}} \right. \kern-\nulldelimiterspace} {\text{L}}}} \right) \times {\text{Fast insulin level}}{{\left( {{{{\mu U}} \mathord{\left/ {\vphantom {{{\mu U}} {{\text{mL}}}}} \right. \kern-\nulldelimiterspace} {{\text{mL}}}}} \right)} \mathord{\left/ {\vphantom {{\left( {{{{\mu U}} \mathord{\left/ {\vphantom {{{\mu U}} {{\text{mL}}}}} \right. \kern-\nulldelimiterspace} {{\text{mL}}}}} \right)} {22.5}}} \right. \kern-\nulldelimiterspace} {22.5}}$$

### Transcriptomics

Total RNA from liver were extracted from 6 random-chosen mice using an RNA isolation kit (Cat. AM1560, Ambion, Austin, TX, US) according to the manufacturer’s protocol of extracting total RNA. After quality and integrity checks, RNA was used for library construction with TruSeq Stranded mRNA Sample Prep Kit (Cat. RS-122-2101, Illumina, San Diego, CA, US). Raw reads of RNA were generated by the Illumina HiSeq^™^ X Ten platform. Reads containing ploy-N and low-quality reads were removed. Clean reads were mapped to NCBI database (GRCm39) using HISAT2 [18].

### Bioinformatic analysis

Fragments per kb per million reads (FPKM) value [19] of gene reads was calculated. DESeq2 were applied to standardize and analyze mRNA expression levels. Threshold for differentially expressed genes (DEGs) were defined as *p* < 0.05 and foldchange >1.5. Principal component analysis (PCA) was used to analyze the relationship between transcriptome of samples. For functional enrichment, Gene Ontology (GO) database and Kyoto Encyclopedia of Genes and Genomes (KEGG) database were used to annotate and analyze identified DEGs.

### Quantitative real-time PCR (RT-qPCR)

Total RNA was extracted using RNA Easy Fast Tissue/Cell Kit (Cat. DP451, Tiangen Biotech, Beijing, China) according to the manufacturer’s instructions. Tissue was homogenized with lysis buffer, the suspensions were filtered through a gDNA eraser column set and an RNase-free spin column. RNA solutions were obtained after column elution with RNase-free double-distilled water. Reverse transcription was performed using HiScript III RT SuperMix for qPCR (Cat. R323-01, Vazyme, Nanjing, China) with 0.5 ng RNA per reaction. RT-qPCR was performed on an Applied Biosystems 7500 Real-Time PCR instrument (Thermo Fisher Scientific) using GoTaq^®^ qPCR Master Mix Kit (Promega Biotech, Madison, WI, US). Primers used in RT-qPCR are listed in Additional file 1: Table S2. The RNA expression levels were normalized to *Gapdh* and quantitated by 2^−ΔΔ*CT*^ method.

### Immunohistochemistry (IHC) examinations

Glycogen synthase (GYS) expression levels were assessed by IHC staining. Liver slices were incubated with anti-GYS primary antibody (Cat. 3886, Cell Signaling Technology) at 4 °C overnight, then incubated with HRP-conjugated goat anti-rabbit IgG secondary antibody (Cat. PV-6000, ZSGB-Bio, Beijing, China) for 20 min and stained with 3,3 N-Diaminobenzidine tertrahydrochloride (DAB) and counterstained with hematoxylin.

### Western blot analysis

Total protein was extracted from liver and adipose tissues using a Total Protein Extraction kit (Cat. KGP250, KeyGen Biotech, Nanjing, China). Protein samples were run in polyacrylamide gels and transferred onto polyvinylidene fluoride membranes (Cat. IPVH00010, Millipore, Bedford, MA, US). After 1 h of blocking at room temperature, membranes were incubated with primary antibodies: PI3K p85 (Cat. 4257, Cell Signaling Technology), p-PI3K (phosphorylation sites: p85 Tyr458/p55 Tyr199, Cat. 4228, Cell Signaling Technology), AKT (Cat. 4685, Cell Signaling Technology), p-AKT (phosphorylation site: Thr308, Cat. 13038, Cell Signaling Technology), and β-actin (Cat. 4970, Cell Signaling Technology) overnight at 4 °C. Membranes were then incubated with HRP-conjugated AffiniPure goat anti-rabbit IgG secondary antibody (Cat. SA00001-2, Proteintech Group, Rosemont, IL, US) for 1 h at room temperature. Blots were visualized using an ultra-sensitive enhanced chemiluminescence reagent (Cat. PE0010, Solarbio). The protein expression levels were analyzed by Image Lab software (Version 3.0, Bio-Rad, Hercules, CA, US) and normalized to β-actin.

### Statistical analysis

All data are shown as means ± standard deviation (SD), where *n* represents the number of biological replicates. Statistical analysis was performed using IBM SPSS Statistics software (Version 23.0, SPSS, Chicago, IL, US). Graphs were composed by GraphPad Prism (Version 7.0, GraphPad Software, San Diego, CA, US). Normally distributed data were analyzed by one-way analysis of variation (ANOVA) with Fisher’s Least Significant Difference (LSD) tests or Dunnett’s test post hoc. Non-normal data were analyzed by nonparametric Kruskal–Wallis test. Comparisons with *p* value < 0.05 were considered statistically significant.

## Results

### LC–MS/MS Analysis of ZSP

**Fig. 1:**
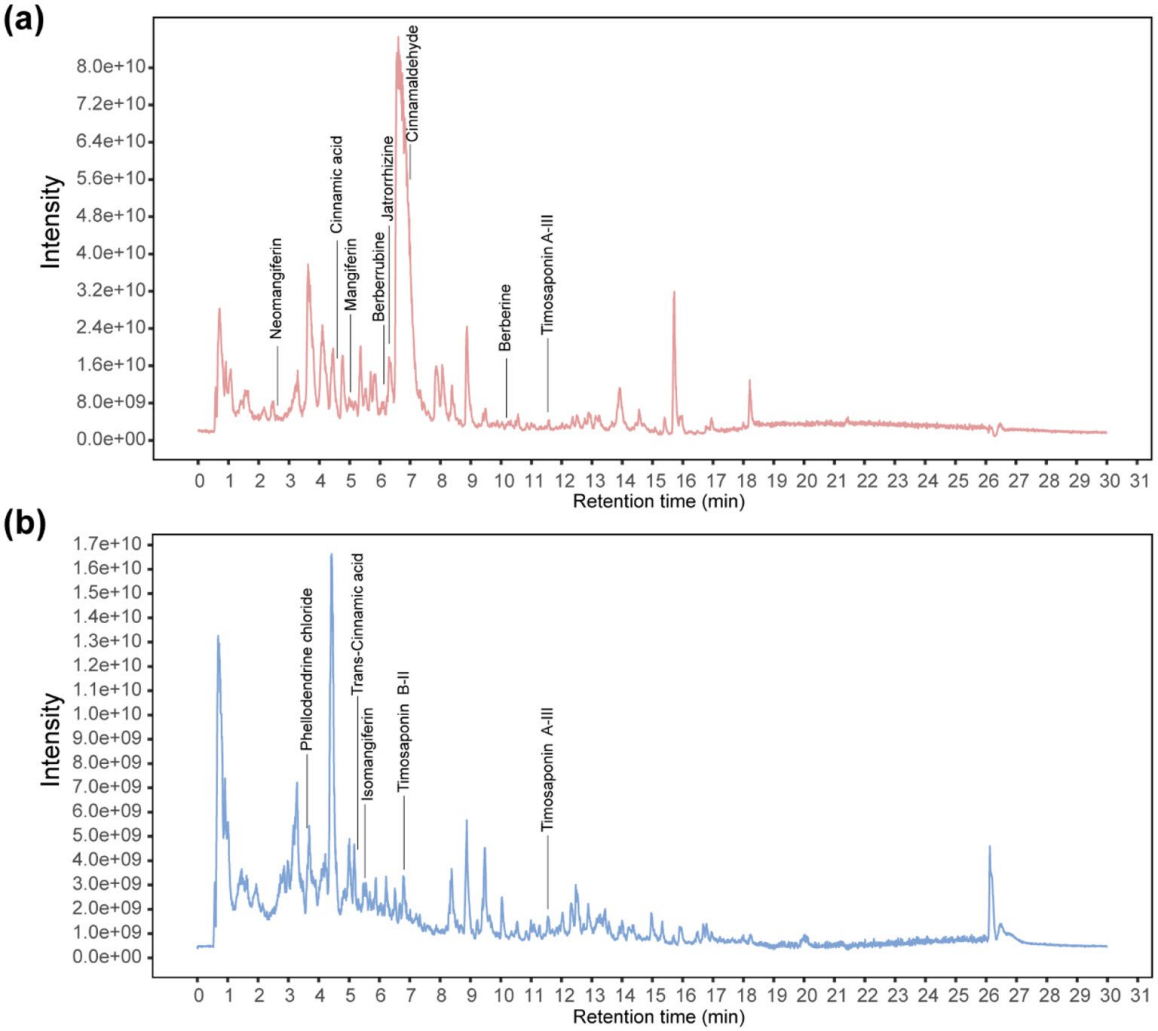
Base peak chromatogram from LC–MS/MS analysis of ZSP under positive ion mode **a** and negative ion mode **b**

The LC–MS/MS analysis identified several characteristic chemicals of the ZSP decoction reported previously [9, 20, 21], including mangiferin, cinnamic acid, berberine and several terpenoids like Timosaponin A-III and Timosaponin B-II. Different chemicals were marked in the base peak chromatogram from positive ion mode (Fig. 1a) and negative ion mode (Fig. 1b). Details of the LC–MS/MS results were listed in Additional file 1: Table S3.

### Safety of ZSP treatment

ZSP treatment did not show acute or chronic toxicity in mice. ZSP treatment had no significant impact on the food or water intake (Additional file 1: Fig. S1a) and did not affect the growth of db/db mice, as body length showed no significant difference between groups (Additional file 1: Fig. S1c). The serum AST and ALT levels were consistent at day 28 or day 40, suggesting ZSP at dose 3.3 g/kg did not negatively impact the liver function of mice (Additional file 1: Fig. S1d).

### ZSP Improves glucose metabolism in db/db mice

**Fig. 2:**
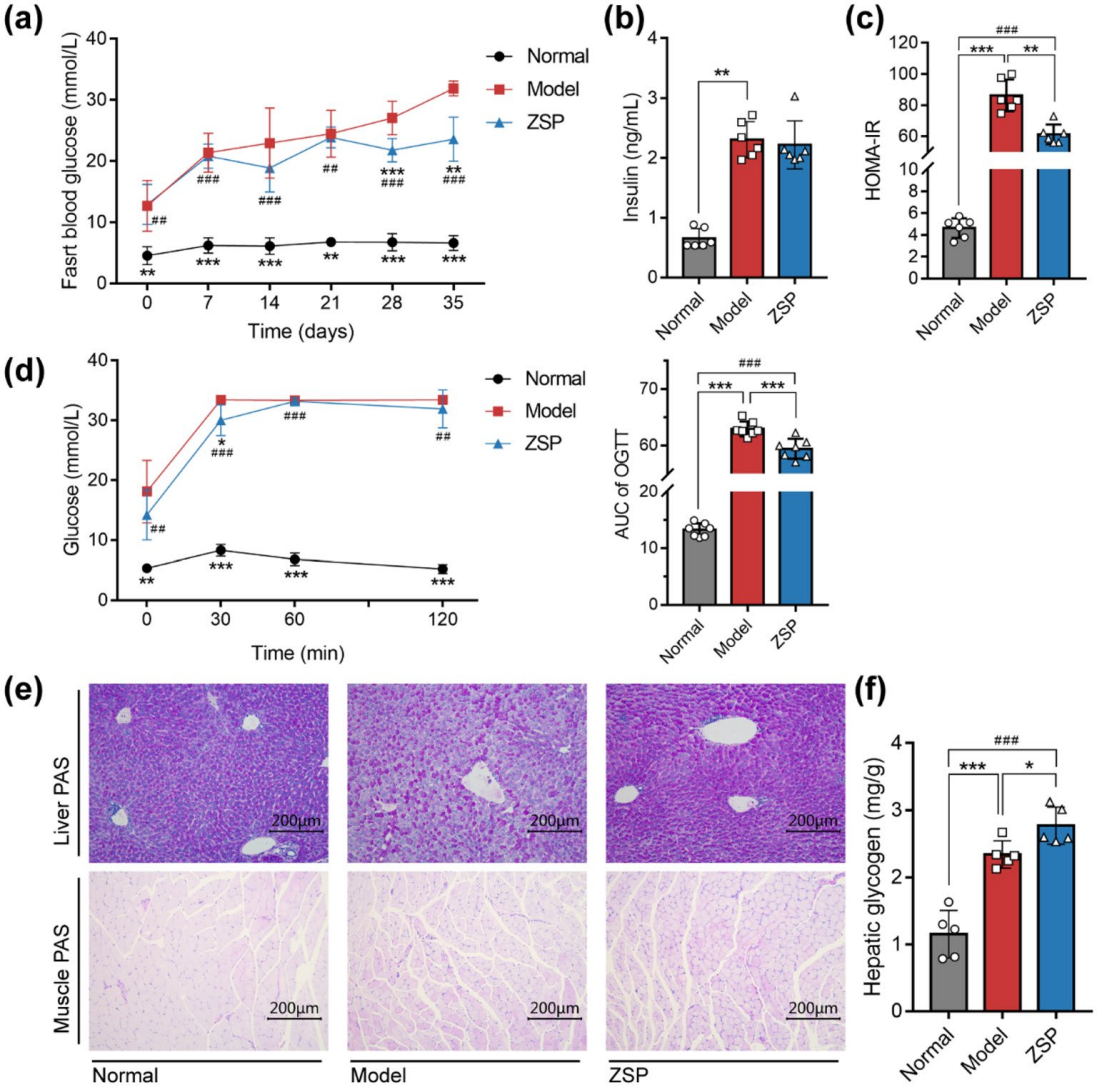
ZSP improves glucose metabolism in db/db mice. **a** Overnight-fasting glucose levels of mice during the treatment (*n* = 7). **b** Fasting serum insulin levels of mice (*n* = 6). **c** HOMA-IR of mice (*n* = 6). **d** Glucose levels from different time points during the OGTT test and the area under curve of each group (*n* = 7). **e** Images of PAS staining of mice liver and muscle tissue. All pictures were taken at 200 × magnification. **f** Hepatic glycogen content of mice (*n* = 5). Normal group: *wt/wt* mice treated with distilled water. Model group: db/db mice treated with distilled water. ZSP group: db/db mice treated with ZSP at 3.3 g/kg/day. All data presented as mean ± SD. * *p* < 0.05, ** *p* < 0.01, *** *p* < 0.001, compared to the model group. # *p* < 0.05, ## *p* < 0.01, ### *p* < 0.001, compared to the normal group

The ZSP treatment group showed significantly lower blood glucose after 28 days (Fig. 2a). ZSP did not alter the serum insulin levels in db/db mice, but did significantly lower the HOMA-IR, suggesting improved insulin sensitivity (Fig. 2b, c). The ZSP group also showed better performance in the OGTT experiment and showed a significant lower AUC value (Fig. 2d). PAS staining indicated that the ZSP treatment improved the glycogen content in the liver, but not the muscle tissue (Fig. 2e), which was corroborated by the glycogen content assay (Fig. 2f).

### ZSP reduced obesity and improved lipid metabolism in db/db Mice

**Fig. 3:**
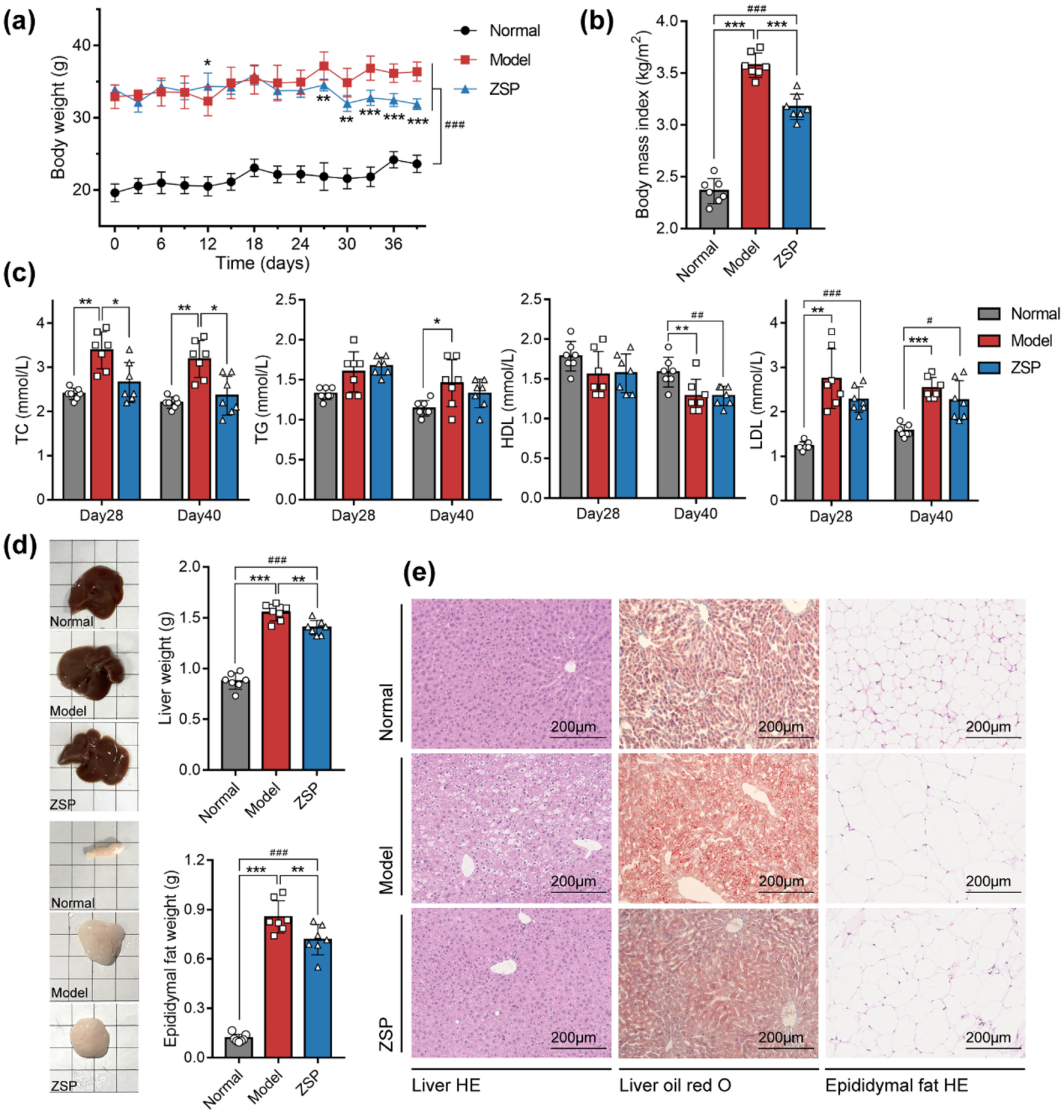
ZSP reduced obesity and improved lipid metabolism in db/db mice. **a** Body weight of mice over the course of treatment (*n* = 7). **b** Body mass index of mice at the end of treatment (*n* = 7). **c** Serum lipid content measured at day 28 and day 40 (*n* = 7). **d** Pictures and weights of liver and epididymal fat of mice (*n* = 7). **e** Images of H&E staining of liver and epididymal fat and oil red O staining of liver, all pictures were taken at 200 × magnification. Normal group: *wt/wt* mice treated with distilled water. Model group: db/db mice treated with distilled water. ZSP group: db/db mice treated with ZSP at 3.3 g/kg/day. All data presented as mean ± SD. *p < 0.05, **p < 0.01, ***p < 0.001, compared to the model group. #*p* < 0.05, ##*p* < 0.01, ###*p* < 0.001, compared to the normal group

The db/db mice showed significantly higher body weight compared to the *wt/wt* mice. After 26 days of treatment, the ZSP treated group showed notable decrease in BMI (Fig. 3a, b). Mice in the model group had higher serum TC, TG, and LDL levels compared to those in normal group. The serum TC and TG values of mice in ZSP group were not significantly different to those in normal group suggesting that ZSP treatment did reduce serum TC content in db/db mice (Fig. 3c).

ZSP treatment also significantly lowered the liver weight and epididymal fat weight of db/db mice (Fig. 3d). The H&E-stained images showed that ZSP partially restored liver steatosis, and oil red O staining suggested that hepatic lipid accumulation was decreased in the ZSP treatment group. Size of epididymal adipocytes in ZSP group were smaller than those in model group. (Fig. 3e).

### ZSP treatment influences the liver transcriptomes of db/db mice

**Fig. 4:**
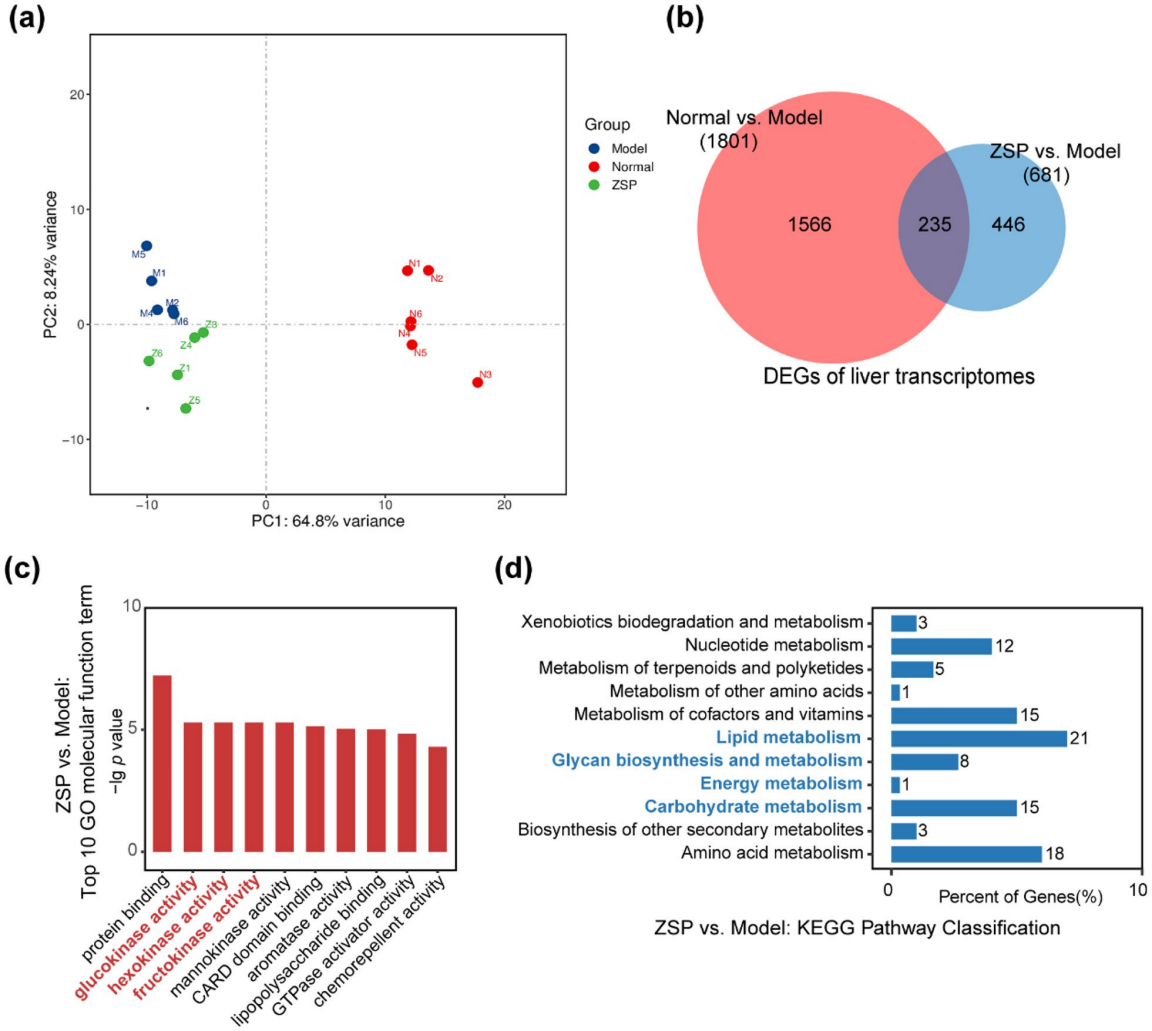
Overview of the transcriptomic results of mice liver. **a** PCA analysis of transcriptome. **b** Venn diagram of DEGs between groups. **c** GO enrichment of molecular function of DEGs between ZSP group and model group. **d** KEGG pathway enrichment of metabolic process of DEGs between ZSP group and model group. Normal group: *wt/wt* mice treated with distilled water. Model group: db/db mice treated with distilled water. ZSP group: db/db mice treated with ZSP at 3.3 g/kg/day

One outlier from both the ZSP group and the model group, were removed due to their inconsistency with other samples from the same group (Fig. 4a). Transcriptomics identified 1801 and 3411 DEGs between the normal and model groups respectively, indicative of a substantial difference in hepatic gene transcription (Fig. 4b). GO enrichment of DEGs from liver transcriptomes revealed ZSP significantly changed glucokinase (GCK), hexokinase (HK) and fructokinase expression (Fig. 4c). HK and GCK catalyze the phosphorylation of hexoses including glucose to hexose-6-phosphate [22–24], the first step in glycolysis. Upregulation of these genes may indicate ZSP enhances glucose utilization through increasing glycolysis. KEGG enrichment analysis revealing a number of DEGs were involved in metabolic processes. Specifically, 21 DEGs were involved in lipid metabolism, 15 DEGs were enriched in carbohydrate metabolism and 8 DEGs were enriched in glycan biosynthesis (Fig. 4d). These results suggest ZSP administration has robust effects on the transcriptome of db/db hepatic cells.

### ZSP enhances hepatic glucose metabolism through PI3K/AKT pathway

**Fig. 5:**
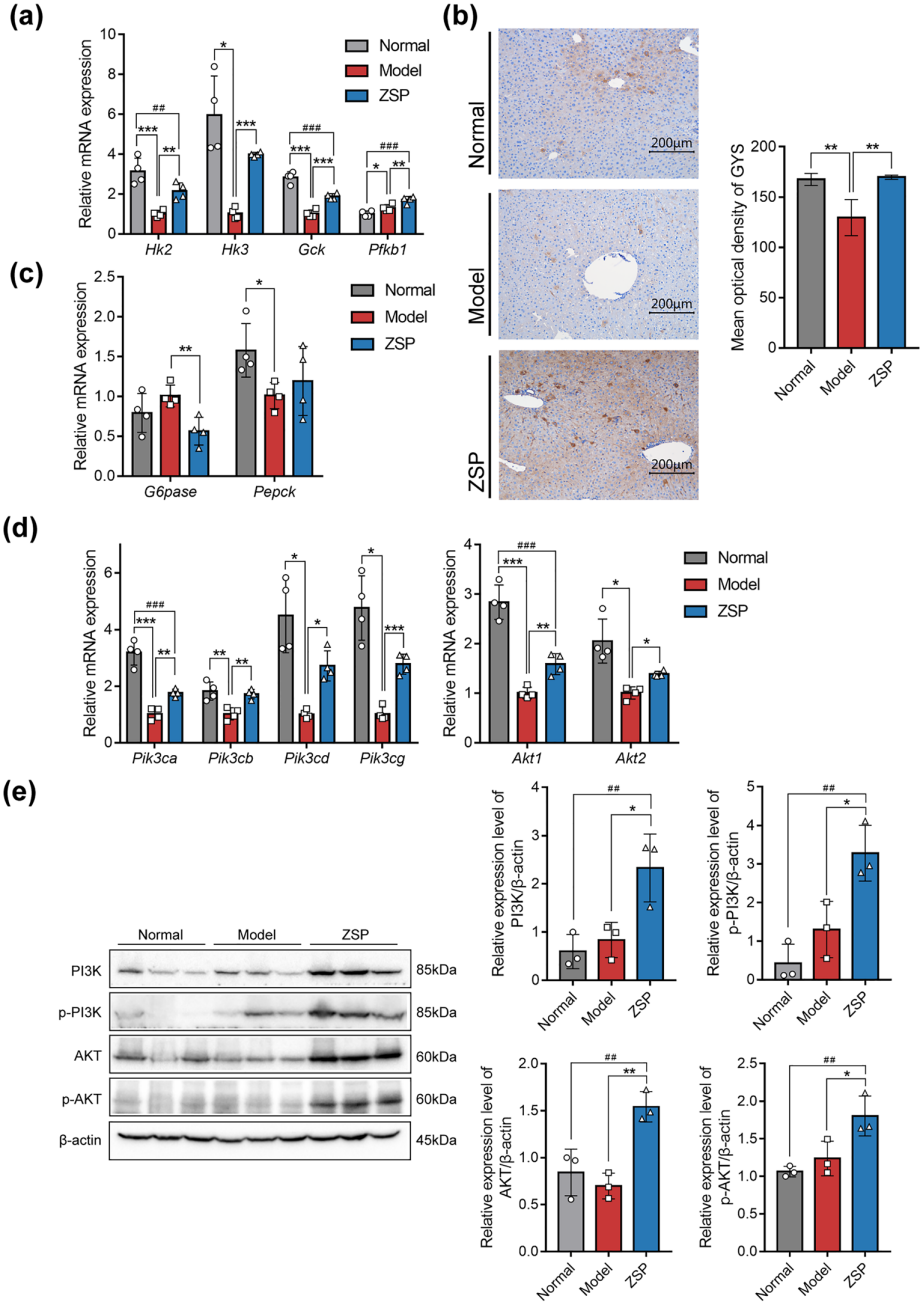
ZSP enhanced hepatic glucose metabolism through PI3K/AKT pathway. **a** Relative mRNA expression levels of *Hk2*, *Hk3*, *Gck* and *Pfkb1* (*n* = 4). **b** IHC staining of GYS and mean optical density of IHC images, pictures were taken at 200 × magnification (*n* = 3). **c** Relative mRNA expression levels of *G6pase* and *Pepck* (*n* = 4). **d** Relative mRNA expression levels of *Pik3ca*, *Pik3cb*, *Pik3cd*, *Pik3cg*, *Akt1* and *Akt2* (*n* = 4). **e** Protein expression levels of PI3K, p-PI3K (phosphorylation sites: p85 Tyr458/p55 Tyr199), AKT and p-AKT (phosphorylation site: Thr308) (*n* = 3). All qPCR results were normalized to model group. Normal group: *wt/wt* mice treated with distilled water. Model group: db/db mice treated with distilled water. ZSP group: db/db mice treated with ZSP at 3.3 g/kg/day. All data presented as mean ± SD. *p < 0.05, **p < 0.01, ***p < 0.001, compared to the model group. #*p* < 0.05, ##*p* < 0.01, ###*p* < 0.001, compared to the normal group

RT-qPCR was used to further explore DEGs of interest. The expression of HK and GCK were significantly lower in model group compared with those of the normal group, ZSP treatment partially restored expression while upregulating the expression of phosphofructokinase (PFK) in treated mice, consistent with the transcriptomic outcomes (Fig. 5a). As hepatic glycogen content was also raised in ZSP group, IHC was used to examine the expression of GYS, a key regulator of glycogen synthesis [25], revealing increased expression in ZSP group (Fig. 5b). ZSP also significantly reduced the mRNA level of *G6pase*, but showed no effects on *Pepck* (Fig. 5c).

In the liver, the expression of *Gck* and *G6pase* are regulated by PI3K/AKT signaling via promoting the translocation of transcriptional factor FOXO1. PI3K/AKT also affects the activity of GYS trough phosphorylating glycogen synthase kinase 3. Since many of the downstream effectors of PI3K/AKT were influenced by ZSP treatment, the levels of PI3K and AKT levels in mice hepatocytes were examined. ZSP increased all genes encoding the catalytic subunit of class I PI3K and the genes encoding both types of AKT (Fig. 5d). The protein levels of PI3K and AKT, as well as their activated forms were all upregulated in the ZSP treated group (Fig. 5e) indicating the improved hepatic glucose metabolism is a product of PI3K/AKT pathway activation.

### ZSP activates PI3K/AKT signaling by influencing GF and RTK expression

**Fig. 6:**
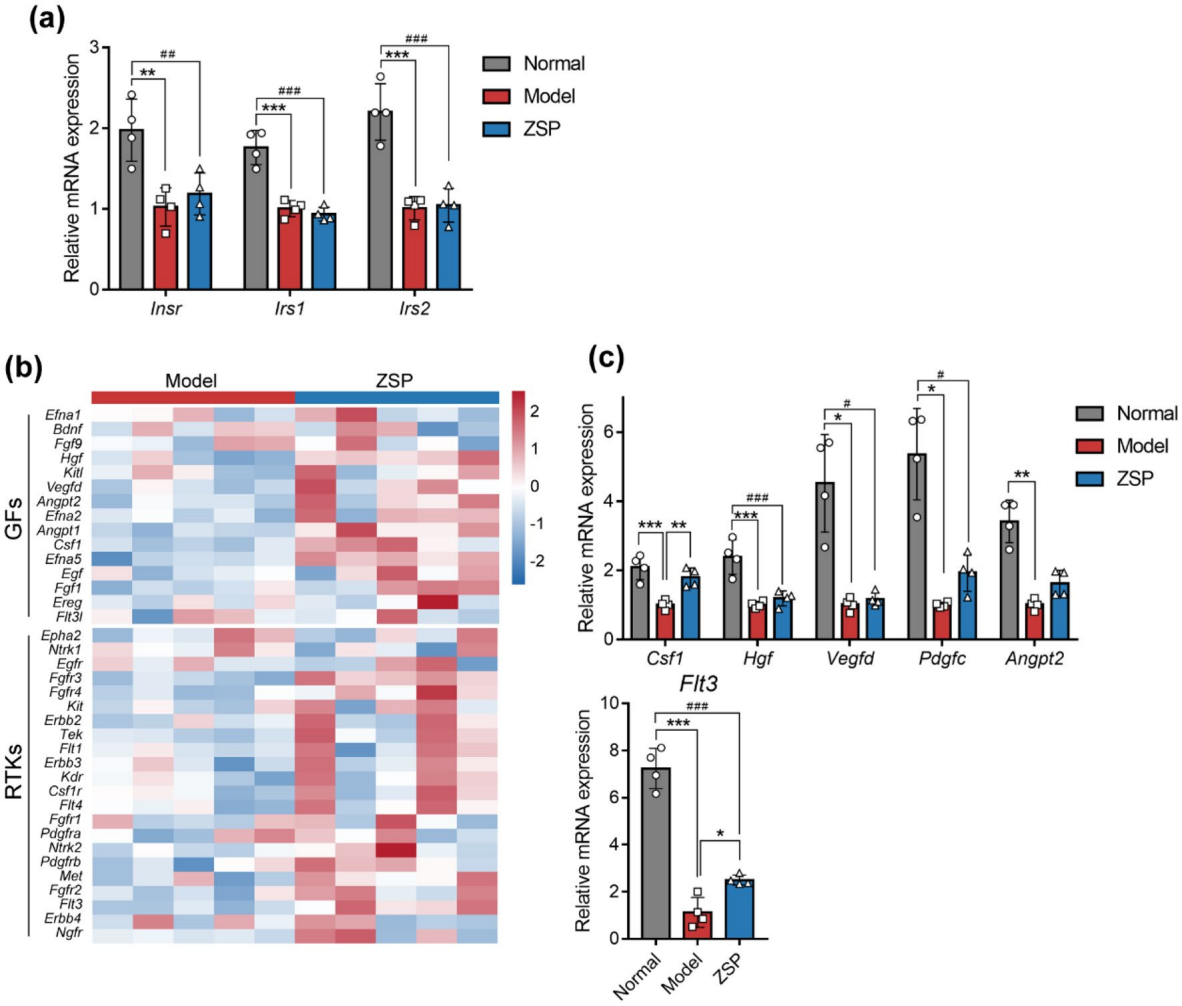
ZSP activates PI3K/AKT signaling through inducing GFs and RTKs. **a** Relative mRNA expression levels of *Insr*, *Irs1* and *Irs2* (*n* = 4). **b** Heatmap of GF and RTK mRNA levels determined by transcriptomic analysis. Color of each box indicates log_2_ fold change for ZSP group vs. model group. **c** Relative mRNA expression levels of *Csf1*, *Hgf*, *Vegfd*, *Pdgfc*, *Angpt2* and *Flt3* (*n* = 4). All qPCR results were normalized to model group. Normal group: *wt/wt* mice treated with distilled water. Model group: db/db mice treated with distilled water. ZSP group: db/db mice treated with ZSP at 3.3 g/kg/day. All data presented as mean ± SD. *p < 0.05, **p < 0.01, ***p < 0.001, compared to the model group. #*p* < 0.05, ##*p* < 0.01, ###*p* < 0.001, compared to the normal group

Unexpectedly, ZSP treatment did not significantly change the mRNA levels of *Insr*, *Irs1* or *Irs2* (Fig. 6a), which are regarded as indicators of insulin activity [26]. Thus, we propose that ZSP may have acted on PI3K through other mechanisms. Comparison of the transcriptomes between db/db mice in the model group and the ZSP group indicate that ZSP had a broad-spectrum promoter effect on many growth factors (GFs) and receptor tyrosine kinases (RTKs) (Fig. 6b). The analysis suggested several significantly altered GFs, including *Csf1*, *Hgf*, *Vegfd*, *Pdgfc* and *Angpt2*, and a significantly upregulated RTK gene *Flt3*. RTKs induce the activation of PI3K, especially the IA subclass [13]. qPCR corroborated ZSP induced increases in the transcriptional expression of *Csf1* and *Flt3*. The average level of *Pdgfc* and *Angpt2* were also higher in the ZSP group, though the difference failed to reach statistical significance (Fig. 6c). This suggests that ZSP may induce the activation of PI3K/AKT via upregulation of GFs and RTKs, particularly *Flt3* and *Csf1*.

## Discussion

In this study, we investigated the effects of ZSP, a traditional Chinese decoction, on a type 2 diabetic mice model. Consistent with previous studies, ZSP treatment was found to significantly attenuate hyperglycemia and obesity in db/db mice, without causing notable toxicity, and with no observed side effects on food or water intake, hepatic or kidney function [8, 9]. Continuing improvement in high-throughput sequencing tests, including the transcriptomics used here, enables robust analysis of disease symptoms on multiple biologically relevant pathways. Through our application of such technology, we were able to identify a range of genes whose expression was influenced by ZSP treatment providing a potential mechanism for ZSP’s effect.

ZSP was found to significantly upregulate the PI3K/AKT pathway, it increased the transcription of four genes catalytic subunit of PI3K: *pik3ca*, *pik3cb*, *pik3cd* and *pik3cg*, as well as increasing the transcription of *Akt1* and *Akt2,* leading to increased protein expression. As PI3K and AKT activation is mediated by phosphorylation of certain amino acids, we examined the phosphorylated PI3K and AKT proteins as well. The results showed that compared to model group, ZSP induced PI3K/AKT signaling in both mRNA and protein levels. However, it is worth noting that the mRNA expression of PI3K and AKT are inconsistent with the protein expression in the normal group, which may be the result of potential different post-transcriptional regulations between the *wt/wt* and db/db mice [27]. Another explain for this phenomenon is that the insulin level in db/db mice is relatively high, and constant stimulation of insulin may also contribute to the elevated PI3K and AKT expression. However, as this study is aiming to investigate the effects of ZSP in a diabetic model, the comparison between treated and untreated db/db mice is of greater relevance here. In db/db mice, the mRNA and protein expression showed coordinate tendencies. Comparison between the treated and untreated group suggests ZSP significantly induced PI3K/AKT activation.

The glucose transported into the liver is metabolized mainly through two ways: glycogen synthesis and glycolysis. After activation, AKT acts on two substrates: glycogen synthase kinase 3 (GSK3) and transcription factor forkhead box O1 (FOXO1). AKT phosphorylates GSK3 and inhibits its activity, thus diminishing the inhibitory effects of GYS and resulting in an increase in glycogen synthesis [28]. AKT also phosphorylates FOXO1 and promotes its exclusion from the cell nucleus, preventing FOXO1 from further influencing gene transcription [29]. This study also revealed that ZSP could raise the rate of hepatic glycogen synthesis possibly by stimulating GYS activity through increased PI3K/AKT signaling. The increase in GYS activity alone is insufficient to raise the glycogen content, as the glycogen content is determined simultaneously by the both synthesis and catabolism. ZSP significantly decreased the transcription of *G6pase*, which is a downstream factor of FOXO1. Glucose-6-phosphatase (G6Pase) dephosphorylates glucose-6-phosphate and generates glucose, which is a rate-limiting step for both gluconeogenesis and glycogenolysis [30]. Therefore, ZSP may increase the hepatic glycogen content by upregulating GYS and downregulating G6Pase.

Aside from being stored as glycogen in the liver, glucose may undergo glycolysis, a step-wise catabolism that provides chemical energy for cell proliferation [31]. ZSP also altered the expression of genes related to glycolysis, specifically, by increasing transcription of *Hk2*, *Hk3*, *Gck* and *Pfkb1* in db/db mice. HKs and GCK catalyze the first step of glycolysis, by phosphorylating glucose to glucose 6-phosphate, whereas PFK metabolizes fructose 6-phosphate into fructose 1,6-bisphosphate. In patients with T2DM, GCK activity is repressed and is correlated with increased fasting glucose levels [32]. Since FOXO1 inhibits the transcription of *Gck* by interfering with corepressor SIN3A [33], and activation of PI3K/AKT suppresses FOXO1, it is possible that the upregulation of GCK by ZSP is mediated by the increased PI3K/AKT signal.

**Fig. 7:**
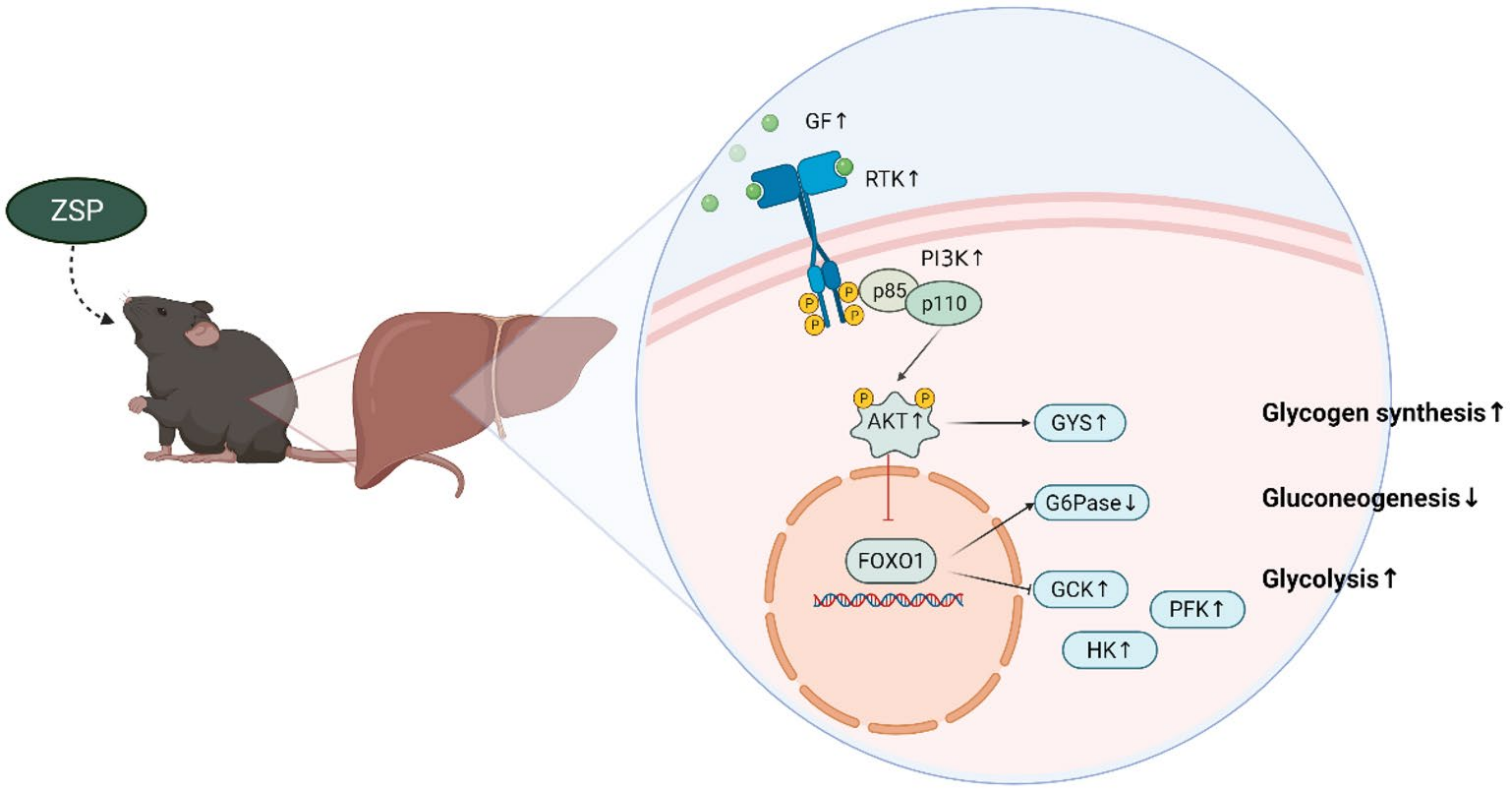
Mechanisms underlying the anti-diabetic effects of ZSP in db/db mice livers. ZSP increases HK, PFK, GCK and GYS, decreased G6Pase. By upregulating GFs and RTKs, ZSP enhances the PI3K/AKT signal transduction in the liver, further increasing glycogen synthesis and glycolysis, and decreasing gluconeogenesis. ↑: upregulated, ↓: downregulated

We also identified RTKs as the upstream factor of ZSP’s effect on PI3K/AKT. The two subclasses of PI3K: class IA and class IB, are typically activated by different signaling protein families. Class IA are activated by RTKs where class IB are activated by G protein-coupled receptors [34]. There are approximately 60 different RTKs encoded by the human genome [35], responsible for communicating extracellular signals by binding with a range of possible ligands including insulin, GFs, and chemokines [36]. The transcriptomic analysis found that ZSP broadly upregulated several GFs and RTKs, and qPCR confirmed a significant increase on *Flt3* expression. It was reported that Fms-like tyrosine kinase 3 (FLT3) mediates PI3K/AKT signaling [37] and treating with FLT3 ligand could significantly prevent the progression to diabetes in diabetic NOD mice [38]. Taken together, these results indicate that ZSP may also activate PI3K/AKT by upregulating GFs and RTKs (Fig. 7).

Insulin resistance is a fundamental pathological change in a number of metabolic diseases, including metabolic syndrome [39], polycystic ovary syndrome [40], nonalcoholic fatty liver disease [41], and T2DM [42]. Clinical studies have demonstrated the link between insulin resistance and T2DM, and insulin-sensitizing managements such as dietary control and exercises are proved effective in dealing with metabolic disorders [42]. Insulin-sensitizing is also the paratheatrical mechanism for some T2DM medicines. Metformin enhances insulin sensitivity in the body by promoting insulin receptor activity, increasing glycogen synthesis and promoting the translocation and activity of glucose translator-4 [43, 44]. Glucagon-like peptide-1 receptor (GLP-1R) agonists, such as Liraglutide, Dulaglutide and Exenatide, act on the GLP-1R expressed on pancreatic islets to promote insulin secretion, and suppress macrophage inflammatory response [45, 46]. Dipeptidyl peptidase-4 (DPP-4) degrades GLP-1 and mediates insulin resistance by impairing the activation of AKT [47]. DDP-4 inhibitors, such as gemigliptin, sitagliptin, teneligliptin, and vildagliptin, have now passed clinical approval and are now actively used to treat T2DM [47, 48]. ZSP, and other traditional herbal remedies like it, show exciting potential as complimentary therapy to current treatment strategies for T2DM [49].

In this research we studied the anti-diabetic effects of ZSP on db/db mice, and explored the molecular mechanisms in the liver. This study did not investigate the dose–effect relationship of ZSP which is certainly worth further exploration. Future studies may also focus on the effects of ZSP in other organs, as diabetes has profound effects on the whole body. As ZSP is taken orally, the decoction effects on gut microbiota may also be worth investigating.

## Conclusion

In conclusion, ZSP treatment reduced the diabetic symptoms in db/db mice, and the hypoglycemic effect may be due to its activation of PI3K/AKT pathway. By upregulating GFs and RTKs, ZSP enhances the PI3K/AKT signal transduction in the liver, increasing glycogen synthesis and glycolysis, and decreasing gluconeogenesis. Considering its effectiveness and lack of toxicity, ZSP provides an intriguing candidate for further development of new T2DM therapies.

## Supplementary Information

**Additional file 1 Table S1.** Elution program used in the UPLC system. **Table S2.** Primers used in qPCR analysis. **Table S3.** Characteristic chemicals identified from LC-MS/MS analysis. **figure S1.** ZSP showed no severe toxic effects on mice. **a** Food intake and water intake of mice (*n=7*). **b** Body length of mice (*n=7*). **c** Assessment of liver function of mice (*n=7*). Normal group: *wt/wt* mice treated with distilled water. Model group: db/db mice treated with distilled water. ZSP group: db/db mice treated with ZSP at 3.3g/kg/day. All data presented as mean ± SD. *p < 0.05, **p < 0.01, ***p <0.001, compared to the model group. #*p *< 0.05, ##*p *< 0.01, ### *p *< 0.001, compared to the normal group. *n s*. non-significant.

## Data Availability

The datasets presented in this study can be found in online repositories. The raw reads of transcriptomic data are deposited in Sequence Read Archive (SRA) database (BioProject: PRJNA835670).

## Abbreviations

AKT: Protein kinase B
ALT: Alanine transaminase
ANOVA: Analysis of variation
AST: Aspartate aminotransferase
AUC: Area under curve
BMI: Body mass index
DAB: 3,3 N-Diaminobenzidine tertrahydrochloride
DPP-4: Dipeptidyl peptidase-4
DEG: Differentially expressed gene
ELISA: Enzyme-linked immunosorbent assay
FLT3: Fms-like tyrosine kinase 3
FOXO1: Forkhead box O1
G6Pase: Glucose-6-phosphatase
GCK: Glucokinase
GF: Growth factor
GLP-1R: Glucagon-like peptide-1 receptor
GO: Gene ontology
GSK3: Glycogen synthase kinase 3
GYS: Glycogen synthas
HDL: High-density-lipoprotein
HK: Hexokinase
HOMA-IR: Homeostatic model assessment for insulin resistance
KEGG: Encyclopedia of genes and genomes
LDL: Low-density-lipoprotein
OGTT: Oral glucose tolerance test
PCA: Principal component analysis
PFK: Phosphofructokinase
PI3K: Phosphoinositide 3-kinase
RTK: Receptor tyrosine kinase
SD: Standard deviation
T2DM: Type 2 diabetes mellitus
TC: Total cholesterol
TCM: Traditional Chinese medicine
TG: Triglyceride
ZSP: Zishen Pill
