# Supplementary material for: Zishen Pill alleviates diabetes in Db/db mice via activation of PI3K/AKT pathway in the liver
Source: Chin Med. 2022 Nov 10;17:128. doi: 10.1186/s13020-022-00683-8 (PMC9647929; doi:10.1186/s13020-022-00683-8)
Supplement: Supplementary file 1 — Additional file 1 Table S1. Elution program used in the UPLC system. Table S2. Primers used in qPCR analysis. Table S3. Characteristic chemicals identified from LC-MS/MS analysis. figure S1. ZSP showed no severe toxic effects on mice. a Food intake and water intake of mice (n=7). b Body length of mice (n=7). c Assessment of liver function of mice (n=7). Normal group: wt/wt mice treated with distilled water. Model group: db/db mice treated with distilled water. ZSP group: db/db mice treated with ZSP at 3.3g/kg/day. All data presented as mean ± SD. *p < 0.05, **p < 0.01, ***p <0.001, compared to the model group. #p < 0.05, ##p < 0.01, ### p < 0.001, compared to the normal group. n s. non-significant. [file 13020_2022_683_MOESM1_ESM.docx]

| Time (min) | Flow rate (μL/min) | %A (0.1% formic acid in water) | %B (0.1% formic acid in acetonitrile) |
| --- | --- | --- | --- |
| 0 | 400 | 95 | 5 |
| 3.5 | 400 | 85 | 15 |
| 6 | 400 | 70 | 30 |
| 6.5 | 400 | 70 | 30 |
| 12 | 400 | 30 | 70 |
| 12.5 | 400 | 30 | 70 |
| 18 | 400 | 0 | 100 |
| 22 | 400 | 0 | 100 |
| 25 | 400 | 0 | 100 |
| 26 | 400 | 95 | 5 |
| 30 | 400 | 95 | 5 |

**Aditional file 1: Table S1.** Elution program used in the UPLC system.

| Gene | Forward primer | Reverse primer |
| --- | --- | --- |
| Gapdh | AAGATGGTGAAGGTCGGTGT | GCTTCCCATTCTCAGCCTTG |
| Pik3ca | ATTTGGCTATAAGCGGGAAC | TTGCTAGGTAAGCCTTGTAACAC |
| Pik3cb | TTATGTCCTCGGCATTGGT | AATAAAAGGTACTCGCTCCC |
| Pik3cd | GGAACAGCCATTCTCCATTGAGC | CTGAGCATACATTCACCTCCGAG |
| Pik3cg | CCCCGAGAGCTTTAGAGTTCC | TTGGAGGCCATCACTTTGCATT |
| Akt1 | TGCCCTGGACTACTTGCACT | ATCTTGATGTGCCCGTCCT |
| Akt2 | CACCCTTCAAACCTCAGGTCAC | GTCCAGGCTGTCATATCGGTC |
| Insr | ATGAGTCAGCCAGTCTTCG | GGCTGTCCTTTGGATACCAC |
| Irs1 | AGCCAGTCTTCATCCAGTTGC | AGATCTCCGAGTCAGTCCCAC |
| Irs2 | ACGTCGTCGCCACAGTTCAGA | ACATTTTCCACAGAGGCCGAAT |
| Hk2 | ACCGTGGACTGGACAACCTCA | AGCCAGATCTCTCACCGTCTCA |
| Hk3 | TGGCTATGATGATCCCCGTTG | CCCCACTCCATGTTGATGCAC |
| Gck | CTTATGGCTGCTACTTCGTTC | CTTCTAGTGCAGCAAAAGCG |
| Pfkb1 | ATGAGCTGCCCTATCTCAAGT | GTCCCGGTGTGTGTTCACAG |
| Gys | CCAACAGCGACATGTACCTC | GTCTCTGCAGGTTTCCGGTT |
| G6pase | ACCTCGTCTTCAAGTGGAT | GACTTCCTGGTCCGGTCTCA |
| Pepck | CCTCAGCTGCATAACGGTCT | CTGAGCATTGCCTTCCACGAAC |
| Flt3 | TTACAAACCAAGACCTGCCTGT | GTACGATGATGGCTTTCCCG |
| Angpt2 | CGCTGTATGATCACTTCTACCTC | AGATGCATTTGTCATTGTCCGA |
| Pdgfc | CACTACAGTATTATCATGCCACA | TCTGGCTCTAGGTACCGAA |
| Vegfd | CTCTTACCTCCAGGAACCCAC | CTCCAGACTTTCTTTGCACTCA |
| Csf1 | CTCTAGCCGAGGCCATGTGGA | GACTAGGATGATGCCCGGCAC |
| Hgf | GAATTCCCTGTCAGCGTTGG | CCTTGCATTTGAAGTTCTCGG |

**Aditional file 1: Table S2.** Primers used in qPCR analysis.

| Chemicals | Molecular formula | Class | | Retention time |
| --- | --- | --- | --- | --- |
| Jatrorrhizine | C20H20NO4+ | Alkaloids | 379.424 | |
| Berberrubine | C19H15NO4 | Alkaloids | 377.934 | |
| Berberine | C20H18NO4+ | Alkaloids | 614.654 | |
| Isomangiferin | C19H18O11 | Xanthones | 235.603 | |
| Epiberberine | C20H18NO4+ | Alkaloids | 376.426 | |
| Tetrahydroberberine THB | C20H21O4N | Alkaloids | 1766.97 | |
| Demethyleneberberine | C19H18NO4+ | Alkaloids | 330.269 | |
| Neomangiferin | C25H28O16 | Xanthones | 160.69 | |
| Cinnamic acid | C9H8O2 | Phenylpropanoids | 266.586 | |
| Mangiferin | C19H18O11 | Xanthones | 303.905 | |
| Cinnamaldehyde | C9H8O | Phenylpropanoids | 418.68 | |
| Timosaponin A-III | C39H64O13 | Terpenoids | 694.654 | |
| Timosaponin B-II | C45H76O19 | Terpenoids | 408.112 | |
| Isomangiferin | C19H18O11 | Xanthones | 332.169 | |
| Phellodendrine chloride | C20H24NO4+.Cl- | Alkaloids | 220.68 | |
| Trans-Cinnamic acid | C9H8O2 | Phenylpropanoids | 328.467 | |

**Aditional file 1 Table S3.** Characteristic chemicals identified from LC-MS/MS analysis.


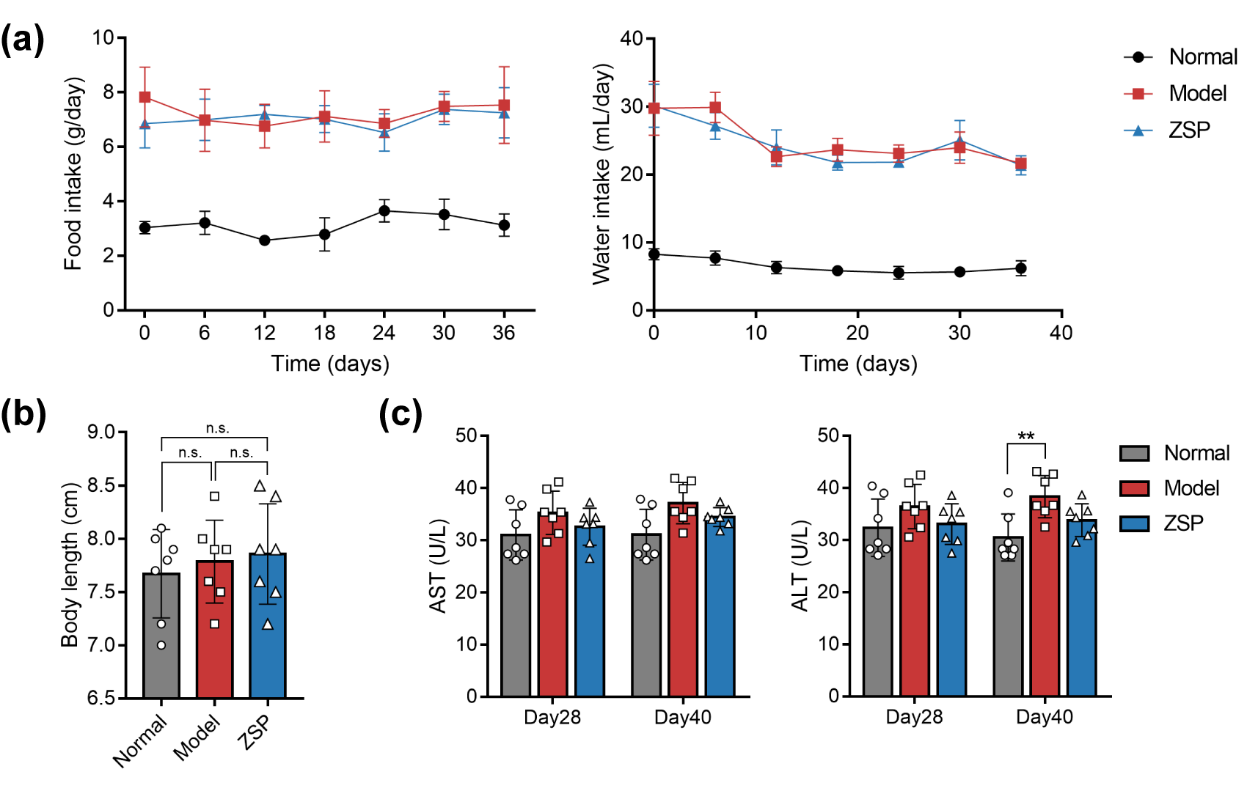


**Aditional file 1: figure S1.** ZSP showed no severe toxic effects on mice. (a) Food intake and water intake of mice (*n=7*). (b) Body length of mice (*n=7*). (c) Assessment of liver function of mice (*n=7*). Normal group: *wt/wt* mice treated with distilled water. Model group: db/db mice treated with distilled water. ZSP group: db/db mice treated with ZSP at 3.3g/kg/day. All data presented as mean ± SD. * *p* < 0.05, ** *p* < 0.01, *** *p* <0.001, compared to the model group. # *p* < 0.05, ## *p* < 0.01, ### *p* < 0.001, compared to the normal group. n.s. non-significant.
